# Phosphodiesterase-1 Inhibitory Activity of Two Flavonoids Isolated from *Pistacia integerrima* J. L. Stewart Galls

**DOI:** 10.1155/2015/506564

**Published:** 2015-04-05

**Authors:** Abdur Rauf, Muhammad Saleem, Ghias Uddin, Bina S. Siddiqui, Haroon Khan, Muslim Raza, Syeda Zehra Hamid, Ajmal Khan, Francesco Maione, Nicola Mascolo, Vincenzo De Feo

**Affiliations:** ^1^Institute of Chemical Sciences, University of Peshawar, Peshawar, Khyber Pakhtunkhwa 25120, Pakistan; ^2^H. E. J. Research Institute of Chemistry, International Center for Chemical and Biological Sciences, University of Karachi, Karachi 75270, Pakistan; ^3^Department of Pharmacy, Abdul Wali Khan University, Mardan 23200, Pakistan; ^4^Department of Pharmacy, University of Naples Federico II, 80131 Naples, Italy; ^5^Department of Pharmacy, University of Salerno, Fisciano, 84084 Salerno, Italy

## Abstract

*Pistacia integerrima* is one of twenty species among the genus *Pistacia*. Long horn-shaped galls that develop on this plant are harvested and used in Ayurveda and Indian traditional medicine to make “karkatshringi”, a herbal medicine used for the treatment of asthma and different disorders of respiratory tract. However, until now, the molecular mechanisms of action of “karkatshringi” and its chemical characterization are partially known. This study deals with the isolation and characterization of the active constituents from the methanolic extract of *P. integerrima* galls and it was also oriented to evaluate *in vitro* and *in silico* their potential enzymatic inhibitory activity against phosphodiesterase-1 (PDE1), a well-known enzyme involved in airway smooth muscle activity and airway inflammation. Our results showed that the methanolic extract of *P. integerrima* galls and some of its active constituents [naringenin (**1**) and 3,5,7,4′-tetrahydroxy-flavanone (**2**)] are able *in vitro* to inhibit PDE1 activity (59.20 ± 4.95%, 75.90 ± 5.90%, and 65.25 ± 5.25%, resp.) and demonstrate *in silico* an interesting interaction with this enzymatic site. Taken together, our results add new knowledge of chemical constituents responsible for the biological activity of *P. integerrima* and contextually legitimate the use of this plant in folk medicine.

## 1. Introduction

The importance of medicinal plants and their derivate in the treatment of various ailments is well known. However, many ethnopharmacological uses are yet to be justified scientifically for a rational and safe use. In this context, plants belonging to* Pistacia* genus (family Anacardiaceae) have attracted the attention of botanists, chemists, and pharmacologists since long time ago [1].

*P. integerrima* J. L. is one of twenty species among the genus* Pistacia* distributed in Himalaya as well as in various regions of Pakistan and India. This plant grows at an altitude of ~3000 meters reaching a height of ~18 meters [2, 3].

Long horn-shaped galls that develop on this plant are harvested and used in Ayurveda and Indian traditional medicine to make “karkatshringi,” an herbal medicine used for the treatment of various ailments including asthma and different disorders of respiratory tract [4–8].

In line with these observations, recent investigations have also demonstrated that essential oils and galls aqueous extract of* P. integerrima* possess an antiasthmatic activity due to the inhibition of histamine release, 5-lipoxygenase activity, and mast cell stabilization [9, 10]. The main constituents isolated from different parts of* P. integerrima* are represented by triterpenoids, sterols, and phenols [11, 12].

On this basis, this study deals with the isolation and characterization of the active constituents from the methanolic extract of* P. integerrima* galls and it was also oriented to evaluate their potential enzymatic inhibitory activity against phosphodiesterase-1 (PDE1), a well-known enzyme involved in airway smooth muscle activity and airway inflammation [13, 14]. To clarify this aspect, here we have also performed molecular docking studies of these compounds on PDE1 binding site.

## 2. Materials and Methods

### 2.1. Reagents

Snake venom phosphodiesterase I (P4631), caffeine (C0750), and* bis*-(*p*-nitrophenyl) phosphate (N3002) were purchased from Sigma-Aldrich Co. (Milan, Italy). Organic solvents such as* n*-hexane, chloroform, ethyl acetate, and* n*-butanol were obtained from Suzhou Ausun Chemical Co., Lit. (Suzhou, China). Unless otherwise stated, all the other reagents were from Searle Pakistan Ltd. (Karachi, Pakistan).

### 2.2. Plant Material

*P. integerrima* was collected from Murree Hills (Pakistan) and it was successively identified by Professor Rashid A. (Department of Botany, University of Peshawar, Pakistan). A voucher specimen (number 20037) was deposited at Department of Botany, University of Peshawar, Pakistan.

### 2.3. Extraction and Isolation

**Figure 1:**
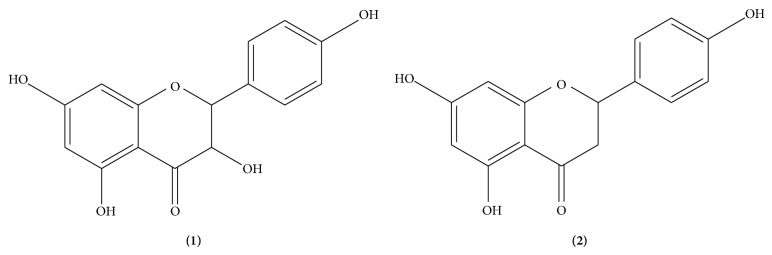
Structure of naringenin (**1**) and 3,5,7,4′-tetrahydroxy-flavanone (**2**).

Coarsely powdered galls of* P. integerrima* (~14 kg) were subjected to maceration at room temperature for 7 days with methanol/water (50 : 50, v/v). The residue was heated under pressure in a water bath at 40°C for further thickening to yield the crude extract (~600 g) and then subjected to chromatography over silica gel and finally eluted with a mixture of methanol/chloroform (100 : 0→0 : 95). The resulting fractions were combined in 10 different subfractions (PS-1 to PS-10) on the basis of TLC. Subsequently, fraction PS-10 (obtained at the elution step 100 : 0→5 : 99, methanol/chloroform) was subjected to repeated chromatography and TLC column in order to obtain the isolated compounds** 1** and** 2** (Figure 1) and fraction PS8 (obtained at the elution step 100 : 0→5 : 99, *n*-hexane/ethyl acetate) led to the isolation of some known constituents such as *β*-sitosterol (compound** 3**), *β*-stigmasterol (compound** 4**), and pistagremic acid (compound** 5**). Their structures were established by comparing their spectral data and physical constants with data already present in literature [15–17].

### 2.4. Phosphodiesterase I Inhibition Assay

Activity against snake venom phosphodiesterase I was assayed by using the reported method [18] with the following modifications: 33 mM Tris-HC1 buffer pH 8.8, 30 mM Mg-acetate with 0.742 mU/well of PDE1 and 0.33 mM* bis*-(*p*-nitrophenyl) phosphate as substrate (vehicle). EDTA (0.2 mM) was used as positive controls [19, 20]. After 30 min incubation of the enzyme with the test samples (0.2 mg/mL and 0.2 mM for methanolic fraction and compounds** 1**–**5,** resp.), enzyme activity was monitored spectrophotometrically at 37°C on a microplate reader (SpectraMax, Molecular Devices, CA, USA) by following the rate (change in OD/min) of release of* p*-nitrophenol from* p*-nitrophenyl phosphate at 410 nm. All assays were conducted in triplicate.

### 2.5. Computational Analysis

The 3D structure of phosphodiesterase protein was downloaded from protein data bank (PDB) with accession code of 3 HMV. The structure was energy minimized through Swiss pdb viewer v4.1.0 program implemented with GROMOS96 force field [21]. The structures of compound** 1**, compound** 2,** and caffeine were drawn in the Chem sketch software [22]. These structures were saved in mol format and, after hydrogen bind addition, energy minimization was carried out through Avogadro software [23]. The docking studies were carried out through Autodock Vina [24] and i-GEMDOCKv 2.1 [25]. For Autodock Vina the solvent molecules were removed from the 3D phosphodiesterase protein and hydrogen atoms addition was calculated [26]. The ligand molecules pdb files were then uploaded in PyRex virtual screening tool [27], connected with Autodock Vina, and then converted into pdbqt format, merging nonpolar hydrogens and adding Gasteiger charges. Charge deficit was spread over all atoms of related residues. Grid center was placed on the active site of the phosphodiesterase protein. We chose coordinates and dimensions along *x*, *y*, and *z* axes of the grid related to the site of presumed pharmacological interest. In particular, we chose a grid box size of 28 × 28 × 28 centered at *x* = −13, *y* = −27, and *z* = −6 with spacing of 1.0 Å between the grid points [28]. Moreover, i-GEMDOCKv2.1 software was also used for docking studies. The best docking conformations were performed twice and implemented with genetic evolutionary algorithm empirical scoring function. Binding pocket was recognized at a distance of 8 Å. Empirical scoring function was estimated as follows: fitness = vdW + Hbond + Elec., where the vdW, Hbond, and Elec. terms represented van der Waal energy, hydrogen binding energy, and electrostatistic energy, respectively [29].

### 2.6. Statistical Analysis

The results obtained were expressed as mean ± S.E.M. For statistical analysis, ANOVA was followed by post hoc Dunnett's test for multiple comparisons. In some case, one sample *t*-test was used to evaluate significance against the hypothetical zero value. Values were considered to be significant at *P* ≤ 0.05.

## 3. Results and Discussion

In our study, we have isolated two flavonoids, naringenin and 3,5,7,4′-tetrahydroxy-flavanone (compounds** 1**-**2**, Figure 1), and three pentacyclic triterpenes (compounds** 3**,** 4,** and** 5**) from the methanolic extract of* P. integerrima* galls. The structures of compounds** 1**-**2** were elucidated by spectroscopic analyses including 1D and 2D NMR experiments (see Supplementary Figures  1, 2, and 3 resp., in Supplementary Material available online at http://dx.doi.org/10.1155/2015/506564), whereas those of compounds** 3**,** 4,** and** 5** were established by comparing their spectral data and physical constants with data already present in literature (Supplementary Figure  3) [15–17].

Compound** 1** was isolated as a pale yellow solid. The molecular formula of compound** 1** was identified as C_15_H_12_O_6_ by HR-EI-MS, EIMS, and ESI spectrum. IR (KBr, *ν*_max⁡_ in cm^−1^): 3599, 1665, 2922, 1591, and 1463. UV *λ*_max⁡_ (nm): 340 and 264. ^1^H NMR (600 MHz, MeOD) *δ*_H_: 5.32 (1H, d, H-2,*j* = 2.0 Hz), 5.78 (1H, d, H-3, *j* = 2.0 Hz), 6.23 (1H, d, H-6, *j* = 8.8 Hz), 6.85 (1H, d, H-8, *j* = 8.8 Hz), 7.11 (1H, d, H-2′, *j* = 8.8 Hz), 6.82 (1H, d, H-3′, *j* = 8.8 Hz), 6.82 (1H, d, H-5′,* j* = 1.2 Hz), and 7.11 (1H, m, H-6, *j* = 10.2) respectively; ^13^C NMR (CDCl_3_, 150 MHz) *δ*_c_: 83.4 (C, C-2), 71.2 (C, C-3), 193.0 (C, C-4), 161.9 (C, C-5), 104.1 (C, C-6), 165.7 (C, C-7), 95.5 (CH, C-8), 158.5 (C, C-9), 104.0 (C, C-10), 130.9 (C, C-1′), 129.9 (CH, C-2′), 117.0 (CH, C-3′), 158.5 (C, C-4′), 117.0 (CH, C-5′), 129.9 (CH, C-6′). The EI showed peak at* m/z* 288, formula C_15_H_12_O_6_. On the basis of the spectral data, compound** 1** was identified as naringenin [30].

Similarly, compound** 2** was isolated as a pale yellow solid. The molecular formula of this compound was identified as C_15_H_12_O_5_ by HR-EI-MS, EIMS, and ESI spectrum (Supplementary Figure 2). IR (KBr, *ν*_max⁡_ in cm^−1^): 3599, 1665, 2922, 1591, and 1463. UV *λ*_max⁡_ (nm): 340 and 264. ^1^H NMR (600 MHz, MeOD) *δ*_H_: 5.95 (1H, d, H-2, *j* = 14.0 Hz), 3.64, 2.74 (2H, dd, *j* = 2.4; 15.1), 6.51 (1H, d, H-6, *j* = 1.8 Hz), 6.63 (1H, d, H-8, *j* = 2.4 Hz), 7.11, (1H, d, H-2′, *j* = 10.2 Hz), 6.82, (1H, d, H-3′, *j* = 10.2 Hz), 6.82 (1H, d, H-5′, *j* = 10.2 Hz), and 7.11 (1H, d, H-6′, *j* = 10.2 Hz), respectively; ^13^C NMR (CDCl_3_, 150 MHz) *δ*: 83.5 (C, C-2), 44.4 (C, C-3), 194.4 (C, C-4), 161.7 (C, C-5), 96.1 (C, C-6), 165.6 (C, C-7), 96.4 (CH, C-8), 158.2 (C, C-9), 104.1 (C, C-10), 130.8 (C, C-1′), 127.8 (CH, C-2′), 116.9 (CH, C-3′), 159.2 (C, C-4′), 116.9 (CH, C-5′), 127.8 (CH, C-6′). The EI showed peak at* m/z* 272, formula C_15_H_12_O_5_. On the basis of the spectral data, compound** 2** was identified as 3,5,7,4′-tetrahydroxy-flavanone.

Naringenin is a hydroxylated flavanone common in grapefruit and other* Citrus* species. This compound is considered to have bioactive effects on human health as antioxidant, free radical scavenger, anti-inflammatory, carbohydrate metabolism promoter, and immune system modulator [31]. 3,5,7,4′-Tetrahydroxy-flavanone is a quite rare compound, found in* Euonymus alatus* (Thunb.) Siebold (Celastraceae) and in* Sygyzium cuminii* L. seeds [32].

Recent investigations have demonstrated that essential oils and galls aqueous extract of* P. integerrima* possess an antiasthmatic activity due to the inhibition of histamine release, 5-lipoxygenase activity, and mast cell stabilization [9, 10]. In addition to these mechanisms, we have taken into account the possibility that the antiasthmatic activity of this plant could be also mediated by the inhibition of phosphodiesterase enzymes (PDEs). Only a few inhibitors of PDE1 have been reported so far and the majority of them, with the exception of rare examples [20], are of synthetic origin.

To this aim, we have tested the methanolic extract and its constituents against snake venom phosphodiesterase I, comparing their inhibitory activities with those of caffeine, a well-known nonselective PDEs inhibitor [33–35].

**Figure 2:**
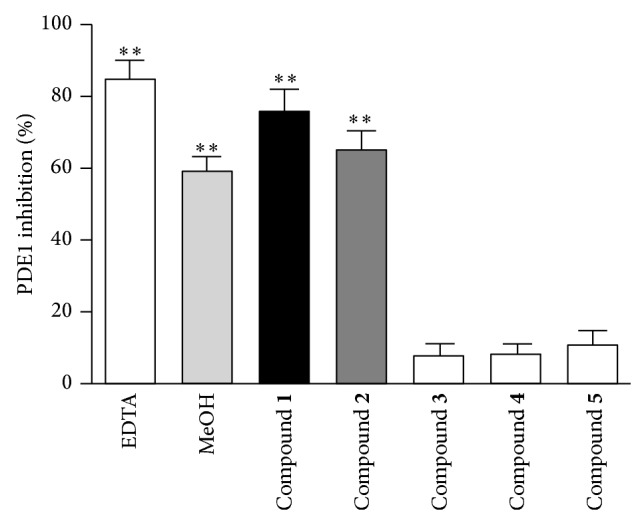
Phosphodiesterase I inhibitory activity (expressed in %) of the methanolic fraction (MeOH, 0.2 mg/mL) of* P. integerrima* and of the isolated compounds** 1**–**5 **(0.2 mM). The assay was performed in presence of positive control EDTA (0.2 mM). Data are expressed as mean ± S.E.M. ^∗∗^ *P* < 0.01 versus vehicle. Data are representative of three different experiments.

**Figure 3:**
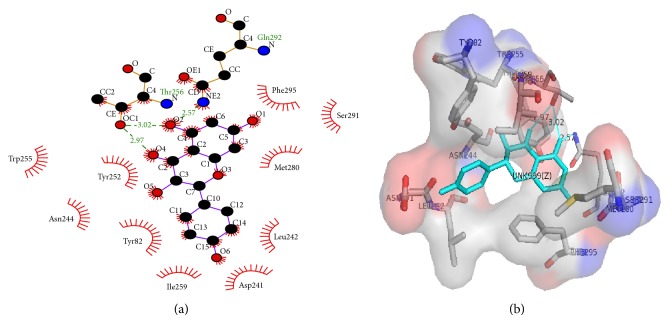
2D (a) and 3D (b) models of naringenin (**1**) in the binding site of PDE1. Green lines indicate hydrogen bonds and the half-moon the hydrophobic interactions.

**Figure 4:**
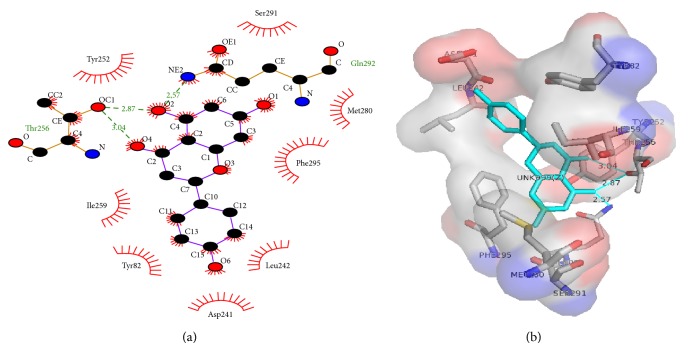
2D (left) and 3D (right) models of 3,5,7,4′-tetrahydroxy-flavanone (**2**) in the binding site of PDE1. Green lines indicate hydrogen bonds and the half-moon the hydrophobic interactions.

**Figure 5:**
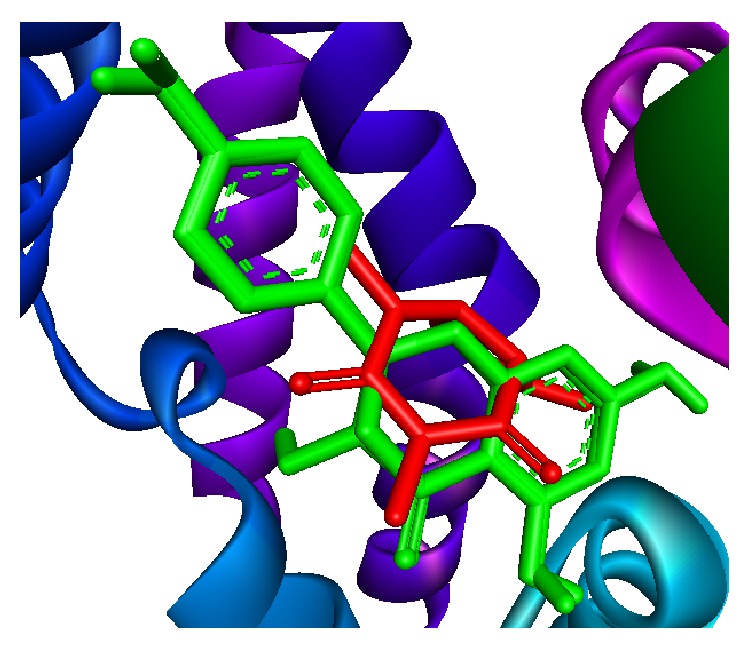
Superimposition of** 1** and** 2** (colored by green) and caffeine (colored by red) in the binding site of PDE1.

Phosphodiesterases (PDEs) are a family of enzymes that catalyze the breakdown of the second messengers cGMP and cAMP [36, 37]. The PDE family has been a focus of drug development in recent years, especially for cardiovascular and airway diseases because of the favorable effects that second messengers have in the vasculature which include increasing vasodilation and decreasing of smooth muscle cells proliferation [38–41]. Recent investigations have also demonstrated that systemic phosphodiesterase inhibitors administration could ameliorate bronchodilation and reduce airway inflammation [13, 14]. The results reported in Figure 2 indicate that methanol extract and compounds** 1** and** 2** possess a significant inhibitory activity against PDE1 enzyme (59.20 ± 4.95%, 75.90 ± 5.90%, and 65.25 ± 5.25% of PDE1, resp.) whereas compounds** 3**,** 4,** and** 5** displayed only partial inhibitory activities (8.05 ± 3.05, 8.20 ± 3.20, and 10.70 ± 4.00%, resp.). The assay was performed in presence of positive control EDTA (85.05 ± 4.95 % of PDE1 inhibitory activity). Successively, we have investigated by docking studies the interaction of compounds** 1** and** 2** with the binding site of PDE1.  Figure 3 shows the interaction of compound** 1** (naringenin) with PDE1 enzyme binding pocket. These interactions including hydrogen bonds formed by Thr256 (with a distance of 3.02 Å and 2.97 Å) and Gln292 residues (with a distance of 2.57 Å) and hydrophobic interactions established by Tyr82, Asp241, Leu242, Asn244, Trp255, Tyr252, Ile259, Met280, Ser291 and Phe295 residues. Similar results were observed for compound** 2**. As shown in Figure 4, the flavonoid establishes hydrogen bonds formed by Thr256 (with a distance of 3.04 Å and 2.87 Å) and Gln292 (with a distance of 2.57 Å) residues and hydrophobic interactions established by Tyr82, Asp241, Leu242, Tyr252, Ile259, Met280, Ser291, and Phe295 residues. Moreover, in order to rationalize the binding mode of compounds** 1** and** 2**, we have used the crystal structure of PDE1 linked to caffeine, a nonselective PDE inhibitor. The docking analysis was carried out through LIGPLOT+ version v.1.4.5, PyMOL version 1.7.2, and discovery studio visualizer version 4.0 software [42, 43]. The analysis of receptor ligand complex based on the hydrogen bond interaction and hydrophobic interaction shows that both compounds displayed a stronger interaction with PDE1 binding site compared with those of caffeine (Figure 5). In particular, the docking result of compounds** 1** and** 2** on PDE1 shows a binding energy of −7.9 kcal/mol and −7.8 kcal/mol, respectively, whereas the total energy was of −110 kcal/mol and −102 kcal/mol. These scores were much lower than that of caffeine (−6.3 kcal/mol; −73 kcal/mol) indicating that the two tested flavonoids possess higher PDE1 activity.

## 4. Conclusions

The results of our study show that the methanolic extract of* Pistacia integerrima* and two of its constituents exhibited a PDE1 inhibitory activity* in vitro*. These evidences were also supported by docking studies suggesting that their established interactions with PDE1 are sufficient to justify the phosphodiesterase inhibitory activity. Taken together, our results add new knowledge of chemical constituents responsible for biological activity of* P. integerrima* and contextually legitimate the use of this plant in folk medicine.

## Supplementary Material

Supplementary Figure 1 and 2: “The structures of compounds 1-2 were elucidated by spectroscopic analyses including 1D and 2D NMR experiments”. The structures of compounds 3, 4, and 5 were established by comparing their spectral data and physical constants with data already present in literature (Supplementary Figure 3).
Supplementary Figure 1. 13C -1H-NMR data (600 MHz, in CDCl3) (A), HMBC (B) and H-H correlations (C) of compound (1).
Supplementary Figure 2. 13C -1H-NMR data (600 MHz, in CDCl3) (A), HMBC (B) and H-H correlations (C) of compound (2).
Supplementary Figure 3. Structure of Pistagremic acid (3), β-Sitosterol (4) and Stigmasterol (5).
